# Using Artificial Neural Network Condensation to Facilitate Adaptation of Machine Learning in Medical Settings by Reducing Computational Burden: Model Design and Evaluation Study

**DOI:** 10.2196/20767

**Published:** 2021-12-08

**Authors:** Dianbo Liu, Ming Zheng, Nestor Andres Sepulveda

**Affiliations:** 1 Department of Biomedical Informatics Harvard University Boston, MA United States; 2 Department of Nuclear Science and Engineering Massachusetts Institute of Technology Cambridge, MA United States

**Keywords:** artificial neural network, electronic medical records, parameter pruning, machine learning, computational burden

## Abstract

**Background:**

Machine learning applications in the health care domain can have a great impact on people’s lives. At the same time, medical data is usually big, requiring a significant number of computational resources. Although this might not be a problem for the wide adoption of machine learning tools in high-income countries, the availability of computational resources can be limited in low-income countries and on mobile devices. This can limit many people from benefiting from the advancement in machine learning applications in the field of health care.

**Objective:**

In this study, we explore three methods to increase the computational efficiency and reduce model sizes of either recurrent neural networks (RNNs) or feedforward deep neural networks (DNNs) without compromising their accuracy.

**Methods:**

We used inpatient mortality prediction as our case analysis upon review of an intensive care unit dataset. We reduced the size of RNN and DNN by applying pruning of “unused” neurons. Additionally, we modified the RNN structure by adding a hidden layer to the RNN cell but reducing the total number of recurrent layers to accomplish a reduction of the total parameters used in the network. Finally, we implemented quantization on DNN by forcing the weights to be 8 bits instead of 32 bits.

**Results:**

We found that all methods increased implementation efficiency, including training speed, memory size, and inference speed, without reducing the accuracy of mortality prediction.

**Conclusions:**

Our findings suggest that neural network condensation allows for the implementation of sophisticated neural network algorithms on devices with lower computational resources.

## Introduction

Machine learning applications for health care can have a great impact on people’s lives. Currently, the possibilities for machine learning in health care include diagnostic systems, biochemical analysis, image analysis, and drug development. One of the most significant challenges in using machine learning for health care applications is that data is usually huge and sparse, requiring important computational resources, especially for overparameterized deep neural networks (DNNs). Consequently, the availability of computational resources to use such tools can limit their widespread use, such as for people who live in low-income countries and for those who want to run diagnostic apps on their own mobile devices.

In this study, we set in-hospital mortality prediction as a case study to explore the various ways of improving efficiency (ie, training speed, memory size, and inference speed) of neural network–based algorithms. Mortality prediction is a well-tried medical machine learning application wherein the mortality of a patient after being transferred to the intensive care unit (ICU) can be predicted based on their vital signs, laboratory tests, demographics, and other factors. Mortality prediction is important in clinical settings because such a prediction can help determine the declining state and need for intervention. We built baseline models with either recurrent neural network (RNN) or dense neural network architectures, based on which we explored efficiency improvements via neural network condensation without sacrificing the prediction accuracy. An RNN is a class of artificial neural networks wherein connections between nodes form a directed graph along a temporal sequence that consider a sequence of input in a recurrent manner. RNNs are widely used in clinical informatics in tasks such as temporal data analysis and clinical natural language processing.

Reduction of complexity and improvement of efficiency of artificial neural networks is an active field of research, wherein a wide range of methods have been explored. One representative example is neural network pruning, wherein a fraction of weights is removed from the trained model and the “lottery ticket” is found when the remaining weight can still be quickly trained with competitive loss and accuracy [1-3]. There are more fancy pruning approaches where the authors use another neural network to learn and conduct the best pruning decisions considering the network to be pruned (ie, the backbone neural network). For example, Lin et al [4] developed a method called runtime neural pruning to model their pruning process as a Markov decision process and use reinforcement learning for training via an additional RNN. Zhong et al [5], on the other hand, used long short-term memory (LSTM) to guide an end-to-end pruning of the backbone neural network. Some other previous works have converted the neural network condensation into an optimization problem where parameters are penalized under some norm [6-9].One RNN-specific condensation method is that instead of embedding information into multiple recurrent layers, we only use one recurrent layer but extend the capacity of the RNN unit (cell) by incorporating more hidden layers within the cell. Dai et al [10] showed that DNNs were inserted between the recurrent layer and the input (masking) layer for each gate in the LSTM to form an LSTM embedded with hidden layers (ie, hLSTM). Such an architecture can, in principle, be more efficient (ie, fewer number of parameters and higher training speed). There is another posttraining condensation method called *quantization*, wherein parameters originally stored in a 32-bit floating point format are forcibly converted to 8 fixed bits [11]. Other methods used for neural network condensation include, but are not limited to, binarization of neural networks [12], knowledge distillation [13], and Huffman coding [14]. In this paper, we describe the use of hLSTM, neural network pruning, and quantization to condense the size of neural networks and increase speed while maintaining their prediction accuracy.

## Methods

### Intensive Care Unit Data

We used the Medical Information Mart for Intensive Care-III (MIMIC-III) critical care database for the implementation of our models [15]. In all, 53,423 distinct hospital adult patients admitted to critical care units between 2001 and 2012 are included in this database. We excluded all neonatal and pediatric patients (aged 18 years or younger at the time of ICU stay) because the physiology of pediatric critical care patients differs significantly from that of adults [16]. We also excluded any hospital admissions with multiple ICU stays or transfers between different ICU units. The final cohort comprised 33,798 unique patients, with a total of 42,276 hospital admissions and ICU stays. Of these 33,798 patients, we defined a test set of 5070 (15%) patient stays. In-hospital mortality was determined by comparing patient date of death with hospital admission and discharge times. The mortality rate within the cohort was 10.9%. The median age of adult patients was 65.8 (SD 11.3) years, and 55.9% (18,893/33,798) patients were male. A mean of 4579 (SD 721.7) charted observations and 380 (SD 215.8) laboratory measurements, as well as other static information, are available for each hospital admission.

**Table 1:** Summary of patient data (N=33,798).

Variable	Value
Mortality during ICU^a^ stay, n (%)	3717 (10.9)
Age in years, median (SD)	65.8 (11.3)
Male participants	18,893 (55.9)

^a^ICU: intensive care unit.

### Data Prepossessing

Data were collected from the MIMIC-III database. Only data from the first 48 hours were used as inputs in our analysis. For the purpose of this study, 76 features were selected for analysis (see examples listed in Textbox 1). Some features may appear multiple times (in different means or conditions) and are thus regarded as *independent* features. We resampled the time series into regularly spaced intervals. If there were multiple measurements of the same variable in the same interval, we used the value of the last measurement. We imputed the missing values using the previous value, if it exists, or a prespecified “normal” [16] value, otherwise. In addition, we added a binary mask input for each variable, indicating the time steps that contain a true (vs imputed) measurement [17]. Categorical variables were encoded using a one-hot vector at each time step. Then, the inputs were normalized by subtracting the mean and dividing it by the SD value. Statistics were calculated per variable after imputation of missing values.

**Examples of the 76 features selected for the analysis.**

- pH
- Fraction-inspired oxygen
- Systolic blood pressure
- Height
- Weight
- Oxygen saturation
- Diastolic blood pressure
- Glucose
- Temperature
- Mean blood pressure
- Capillary refill rate
- Respiratory rate
- Heart rate
- Fraction inspired oxygen
- Glasgow Coma Scale–50

### Performance Metrics

Classification accuracy of all models were measured using area under the receiver operating curve AUROC (also called AUCROC) on the test set. Sizes of model were measured by the number of parameters and sizes of the saved model file. Inference speed was calculated based on time taken to make predictions on test data and was normalized per patient. We used Python 3.6, Keras 2.2.4 with TensorFlow 1.1.2, as the backend for the analysis.

### RNN Model

**Figure 1:**
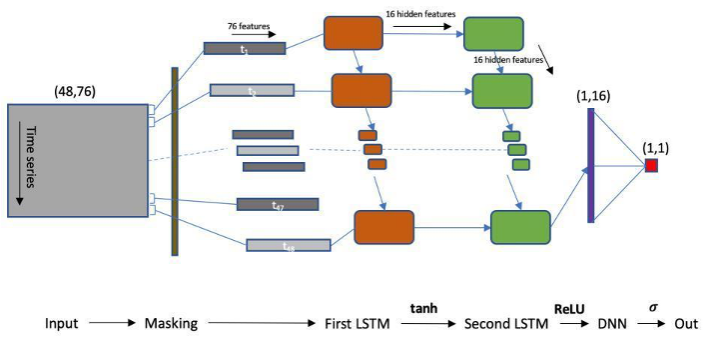
Architecture of recurrent neural network baseline model. DNN: deep neural network; LSTM: long short-term memory; ReLU: rectified linear unit.

Our RNN baseline model is designed as an RNN consisting of a masking layer, two LSTM layers, a dropout layer, and a dense output layer, as shown in Figure 1. We chose two layers of LSTM because, based on a literature review, we identified this structure to be the one with the best performance in the MIMIC-III mortality prediction work [16]. The masking layer masks (skips) the time step for all downstream layers if the values of input tensor at the time step are all equal to zero, which represents missing data for that time step. The first layer of LSTM takes in the original 76 features and generates a 16-feature hidden state based on the hidden state of the previous step and the new incoming observation. Then, such a hidden state is forward to the entrance of the second LSTM layer, which produces another 16-feature hidden state at each step. A dropout layer is followed by the last-step hidden state of the second LSTM layer to prevent complex coadaptations of the neurons. Finally, a dense layer is used to generate a soft 0/1 mortality prediction. The training was conducted using Adam algorithm with a dropout rate of 0.3 between layers and a learn rate of 0.001. In this study, hyperparameters were chosen by grid searching based on performance on the validation set.

### hLSTM Model

Besides pruning upon RNN, we also tried another way by inserting an additional hidden dense layer into the inner gates of LSTM, which we called hLSTM, to improve the “power” of the LSTM. For a traditional LSTM, the inner structure is as follows:



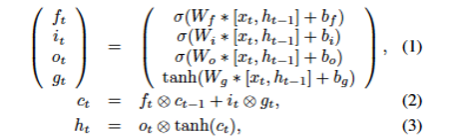



where * is the matrix product; ⊗ is the element-wise product; *W* represents recurrent kernel matrices of the gates; and *b* represents corresponding bias terms. Moreover, *f, i, o, c, x, h* and *c* represent the forget gate, input gate, output gate, vector for cell updates, input, hidden state, and cell state, respectively. Subscript *t* indicates the time step. For hLSTM, the recurrent layer in equation 1 is modified as follows:



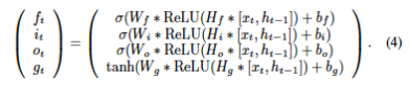



### Feedforward DNN Model

Our baseline feed forward artificial neural network—commonly called DNN—used in this project consists of three fully connected layers, a dropout layer, and an output layer. The fully connected layers have 256, 128, and 64 neurons, respectively, and they use rectified linear unit (ReLU) as the activation function. The dropout layer has a probabilistic dropout rate of 0.5. Sigmoid function was used as activation at the output layer. The loss function used was binary cross-entropy, and the optimization algorithm used was Adam. The baseline DNN model and the pruned DNN model (pDNN) were all trained for 20 epochs, using a batch size of 8. The input into the DNN model has the same feature set as LSTM model but does not consider time series information. The values were calculated by averaging nonmissing values across time steps.

### Neural Network Pruning

All neural network prunings were conducted at the channel level, which means a neuron and all its inputs and outputs were removed from the model if the neuron is pruned. Keras surgeon library in python was used for pruning. In each layer, neurons were pruned if their mean weight across all inputs from the previous layer were below the set quantiles (ie, 25% and 50% in this study). The original model was trained for 1 epoch before pruning and was trained for another 19 epochs after pruning.

### Neural Network Quantization

Quantization was applied on the DNN model post training. Parameters, including weights and activation, originally stored in a 32-bit floating point format were converted to 8 bits using TensorFlow Lite. A uniform quantization strategy was used, as previously described [11]. Considering the range of float point values in the model to be (F_min_; F_max_), all the floating-point values were quantized into the range (0; 255) as 8 bits in a uniform manner, where F_min_ corresponds to 0 and F_max_ corresponds to 255.

The quantization process is



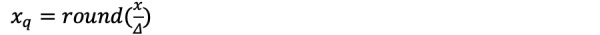



where *x* is the floating-point variable, *x_q_* is the quantized variable, and



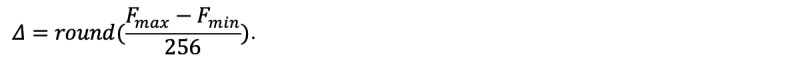



## Results

### Recurrent Artificial Neural Network Condensation: hLSTM and Pruned LSTM

**Figure 2:**
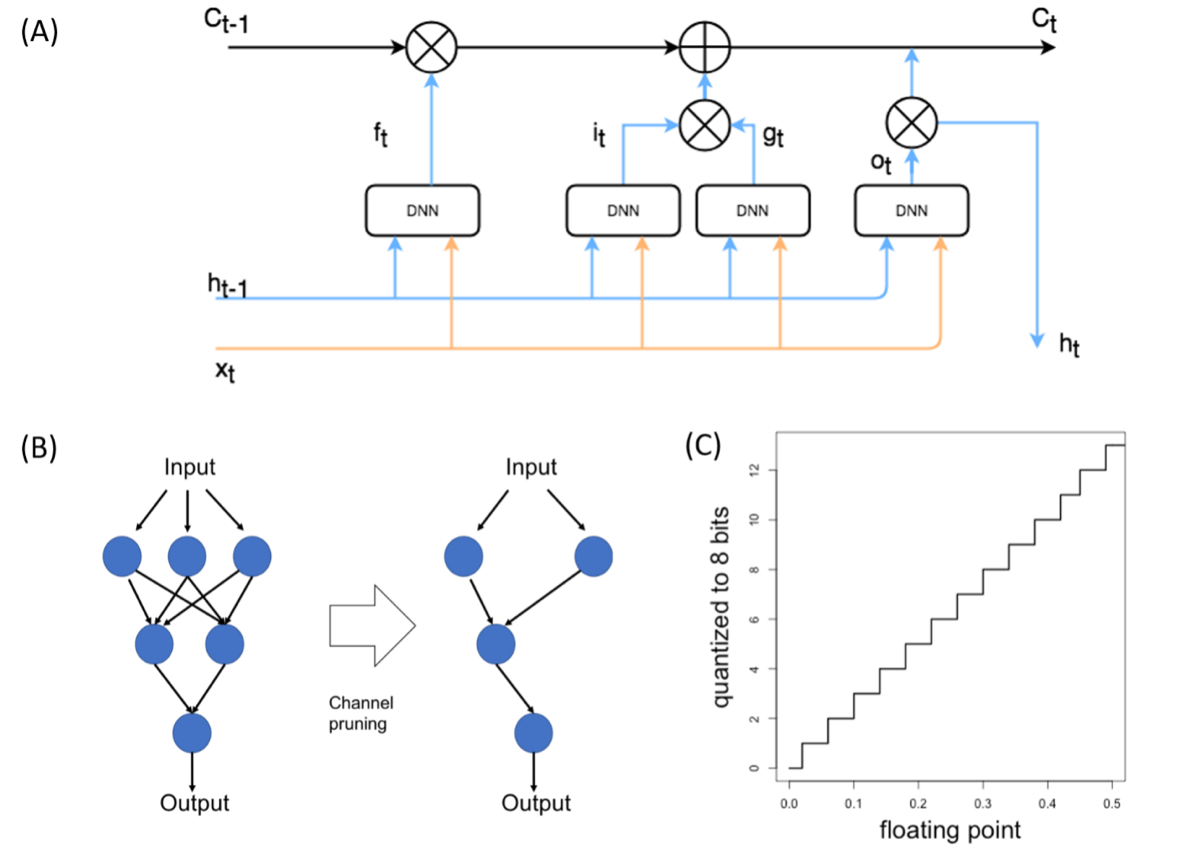
Neural network condensation methods. (A) Hidden-layer long short-term memory (LSTM). Instead of single fix layer nonlinearity for gate control of LSTM, multiple layer neural network with ReLu as activation were used to enhance the gate controls. In this way, fewer layers of LSTMs were needed to build a model with similar performance. (B) A large portion of parameters in artificial neural networks are redundant. We pruned 50% of the channels (neurons) with the lowest weights in each layer to reduce size and complexity of the neural network. (C) Most artificial neuron network implementation in research settings uses 32- or 64-bit floating points for model parameters. We quantized the parameters to 8 bits after training to reduce sizes of the models. DNN: dense neural network.

Recurrent artificial neural networks (or simply, RNNs) are a group of machine learning models widely used in clinical settings that take sequential or time series information as the input. However, training of RNNs and running inference from RNNs are relatively computationally intensive. In order to enable the machine learning algorithms to be used on devices with limited computational power, such as those in high-income countries and on mobile devices, we used three strategies to reduce the storage size of the model and to increase the speed of training and inference (Figure 2).

We built a baseline RNN using two layers of LSTM neurons to predict ICU mortality rates using MIMIC-III dataset [15]. After training, the baseline RNN model achieved a decent performance of AUROC of 0.85 (Table 2).

**Table 2:** Recurrent neural network condensation.

Model	Parameters, n	File size (kb)	Inference (seconds per sample)	Training time (seconds; 20 epochs)	Test AUROC^a^ (last epoch)
Baseline LSTM^b^	8081	129	523	4890	0.836
Pruned LSTM	3273	73	318	4990	0.853
Hidden-layer LSTM	6993	111	254	3000	0.860

^a^AUROC: area under the receiver operating curve.

^b^LSTM: long short-term memory.

The first strategy was to modify the LSTM cell to increase the representation power of each layer. We modified the original neural network structure and added an additional hidden layer into the original LSTM class, wherein one additional layer called “hidden kernel” was inserted between the input kernel and the recurrent kernel (see equation 4). By using this strategy, we replaced the old 2-layer LSTM with only one layer of hLSTM, such that we simplified the overall structure by trying to embed the same quantity of information in this single “condensate” layer.

**Figure 3:**
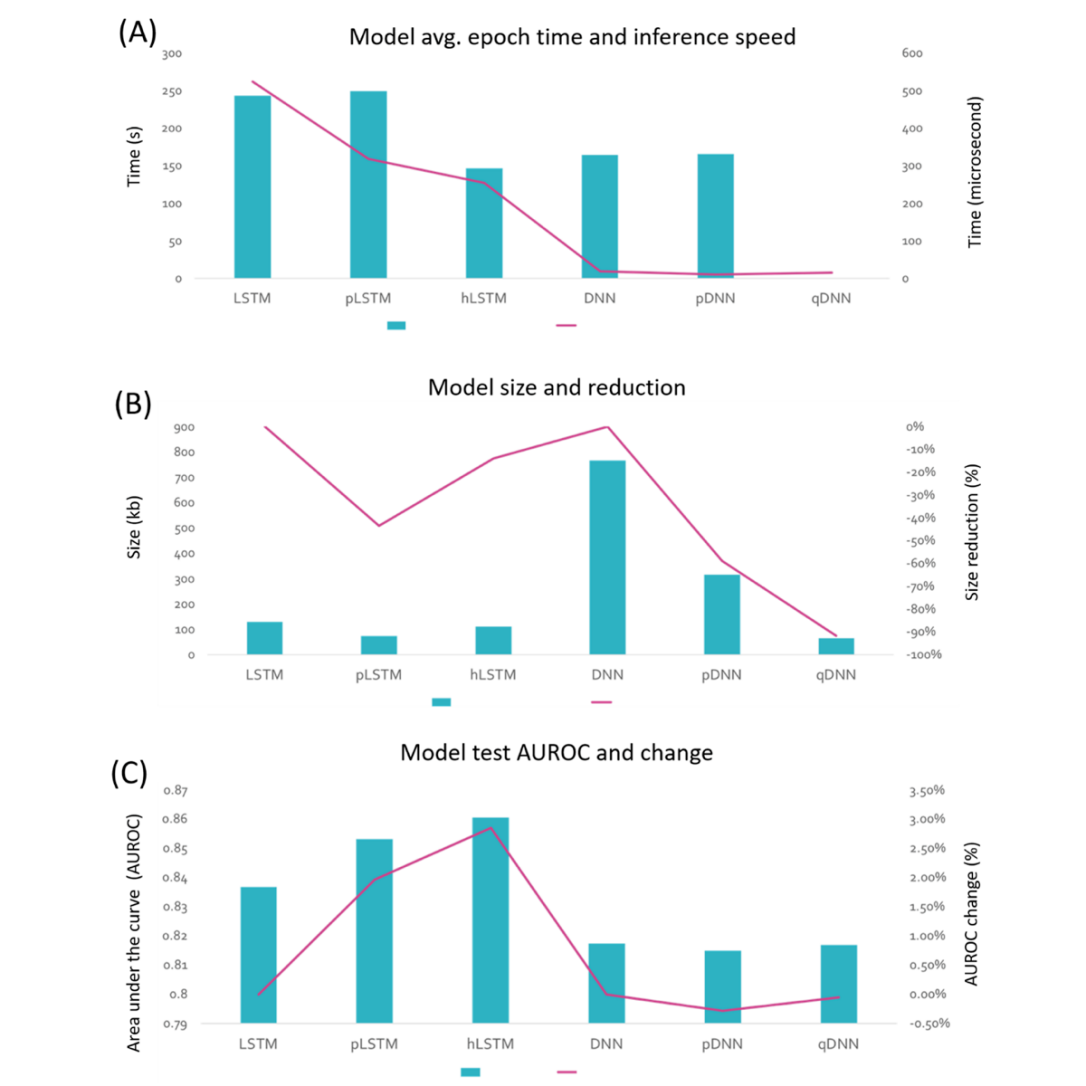
Accuracy, model size, and inference speed of feedforward recurrent and neural networks (RNNs) after different types of condensation. (A) Area under the receiver operating characteristic curve (AUROC) of various models. (B) Various model sizes in memory. (C) Inference speed of various models. Models included the RNN baseline model with two layers of long-term short memory (LSTM), pruned LSTM (pLSTM) model, and one hidden layer inserted in LSTM (hLSTM); deep neural network (DNN) baseline model; pruned DNN (pDNN) model; quantized DNN (qDNN) model.

Both the baseline model and the hLSTM model with only one layer of hLSTM are trained under the same settings. The comparison of AUROC and accuracy is shown in Figure 3. The number of parameters for these two models are listed in Table 2. This simplified model with a single layer of hLSTM beats the baseline model 2-fold in training speed, achieving a 32% reduction in parameter numbers while simultaneously maintaining a higher AUROC at the same time.

**Figure 4:**
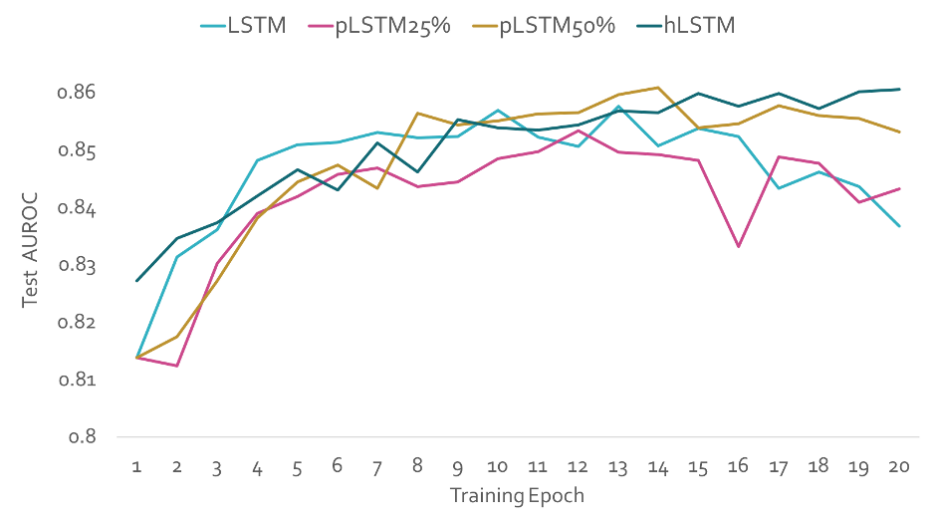
Test area under the receiver operating curve (AUROC) by training epoch for recurrent neural network (RNN) models. Evolution of different RNN models over training epochs on test data. The percentage next to the pruned long short-term memory (pLSTM) model indicates the pruned percentile. hLSTM: hidden-layer long short-term memory; LSTM: long short-term memory.

Another method to condense RNN models is pruning, in which some unessential neurons of the RNN model are removed to minimize model size and increase speed. About 50% of LSTM neurons with lowest weights in each hidden layer were pruned after the first epoch of training. The pruned LSTM only has half of the number parameters of the original LSTM, but it achieves a similar level of accuracy, yielding an AUROC of 0.85 (Figure 4). The inference speed of pruned LSTM also doubled compared with the original LSTM (Table 2).

### Feedforward Neural Network Condensation: Pruning and Quantization

Feedforward neural network, or commonly called DNN if it has multiple hidden layers, is another widely used form of machine learning in clinical settings. We trained DNN with 3 hidden layers, consisting of 256, 128, and 64 neurons in each layer, to enable ICU mortality prediction. The baseline DNN achieved an AUROC of 0.82, using patient data collected within the first 48 hours after admission. We explored two methods to condense the size of the DNN. The first method, called pruning, used the pruning strategy as in RNN; for this purpose, 50% of the channels were pruned after the first epoch of training, the prediction accuracy of the pDNN maintained at the same level as the original DNN, and the inference speed doubled (Table 3). The second strategy involved quantization, which refers to the process of reducing the number of bits that represent a number. In the context of this project, the predominant numerical format used was a 32-bit floating point. We used an after-training-quantization strategy to represent the parameters of the DNN model using 8-bit integers (ie, quantized DNN or qDNN). This method reduced storage size of the DNN model by 5 times without incurring significant loss in accuracy (Table 3). We also compared the overall performances of DNN condensation with those of RNN, as shown in Figure 3.

**Table 3:** Feedforward neural network condensation.

Model	Parameters, n	File size (kb)	Inference (seconds per sample)	Training time (seconds;20 epochs)	Test AUROC^a^ (last epoch)
Baseline DNN^b^	60,929	767	20	3300	0.82
Pruned DNN	27,312	315	10	3310	0.81
Quantized DNN	60,929	64	15	N/A^c^	0.82

^a^AUROC: area under the receiver operating curve.

^b^DNN:

^c^N/A: not applicable.

## Discussion

In this study, we were able to use data from the MIMIC-III database [15] to train in-hospital mortality neural network models with high accuracy and conduct model condensation with different methods to gain efficiency (eg, memory size reduction and increased speed) without compromising accuracy. We implemented different neural network architectures for both RNNs and dense neural networks; thus, our methods can add value in both settings. We pioneered RNN pruning with clinical implementation and our condensation treatments aiming at higher efficiency can be extended to other medical applications using similar data, and probably to nonmedical applications as well. In addition, in medical settings, model calibration is conducted after initial model training. Calibration can be conducted using various training schemes and early stopping strategies. The model condensation method proposed in this study significantly reduces the number of parameters and will help make model calibration easier. The major limitation of the neural network condensation method is that although our proposed method significantly reduces the sizes of different models and their computational costs in training, the final model sizes after condensation are still proportional to the original model sizes. Therefore, if further model size reduction is warranted, a combination of better model design and neural network condensation will be required.

## Abbreviations

AUROC: area under the receiver operating curve
DNN: deep neural network
hLSTM: hidden-layer long short-term memory
ICU: intensive care unit
LSTM: long short-term memory
MIMIC-III: Medical Information Mart for Intensive Care-III
pDNN: pruned deep neural network
qDNN: quantized deep neural network
ReLU: rectified linear unit
RNN: recurrent neural network
